# Future Therapies of Wet Age-Related Macular Degeneration

**DOI:** 10.1155/2015/138070

**Published:** 2015-02-24

**Authors:** Makoto Ishikawa, Daisuke Jin, Yu Sawada, Sanae Abe, Takeshi Yoshitomi

**Affiliations:** Department of Ophthalmology, Akita Graduate University School of Medicine, Akita 010-8543, Japan

## Abstract

Age-related macular degeneration (AMD) is the leading cause of blindness in the elderly population, and the prevalence of the disease increases exponentially with every decade after the age of 50 years. While VEGF inhibitors are promising drugs for treating patients with ocular neovascularization, there are limitations to their potential for improving vision in AMD patients. Thus, future therapies are required to have the potential to improve visual outcomes. This paper will summarize the future strategies and therapeutic targets that are aimed at enhancing the efficacy and duration of effect of antiangiogenic strategies.

## 1. Introduction

Age-related macular degeneration (AMD) is a leading cause of blindness that affects the macular region of elderly individuals [1]. The early phase of AMD is characterized by RPE changes and subretinal deposits (drusen) between the retinal pigmented epithelium (RPE) and Bruch's membrane [2]. The disease can progress to either choroidal neovascularization (CNV), a rapidly deteriorating late form of AMD characterized by new blood vessels that invade the macula (wet AMD), or to geographic atrophy (dry AMD), a slower, late onset form that causes degeneration of the RPE in the macula [3].

It has been known that vascular endothelial growth factor (VEGF) is the most important angiogenic regulator of CNV [4] and a prominent promoter of vascular permeability in AMD [5, 6]. For this reason, VEGF is a key target in treating AMD. * * Current treatment of wet AMD involves the inhibition of VEGF via intravitreal injection of VEGF inhibitors (bevacizumab, pegaptanib, ranibizumab, or aflibercept) [7].

While VEGF inhibitors are promising drugs for treating patients with ocular neovascularization, there are limitations for ameliorating vision in wet AMD patients [8]. Anti-VEGF therapy also requires monthly or bimonthly injections and assessments to determine whether patients have responded to the treatment [8]. These injections and clinical assessments may impose an enormous drain on patients as well as ophthalmologists [8]. Therefore, it is required to develop options that have the potential not only to reduce patient visits and injections, but also to improve visual outcomes by increasing drug effectiveness and lengthening treatment durability [8].

While the VEGF pathways have proven to be a successful target in AMD treatment, there are many other new therapies and approaches in the pipeline, which hold promise for improving the treatment of wet AMD [7]. As our understanding of the molecular mechanisms involved in AMD increases, multiple new treatments are emerging. In this review, we describe the present knowledge concerning the therapeutic targets of AMD and investigational drugs and treatments listed in Table 1.

## 2. PDGF Inhibition

Platelet-derived growth factor (PDGF) stimulates angiogenesis, as well as pericyte recruitment and maturation [9]. Pericytes play an important role in protection of endothelial cells against VEGF inhibition [10, 11]. Consistently, inhibition of PDGF increases endothelial cell sensitivity to anti-VEGF agents [10, 12].

E10030 (Ophthotech Corporation, Princeton, NJ, USA) is an aptamer directed against PDGF and is currently enrolling in parallel phase 3 clinical trials in combination with anti-VEGF injections (ClinicalTrials.gov identifier: NCT01940900). E10030 decreases pericyte density in neovascular vessels and therefore improves the effect of anti-VEGF agents [12].

A phase 1 study of patients (*n* = 22) evaluated intravitreal E10030 in combination with ranibizumab (a phase 1, safety, tolerability, and pharmacokinetic profile of intravitreous injections of E10030 (anti-PDGF pegylated aptamer) in subjects with neovascular age-related macular degeneration, ClinicalTrials.gov identifier: NCT00569140). In the combination group, 59% of patients experienced at least a three-line gain in visual acuity by 12 weeks (a phase 1, safety, tolerability, and pharmacokinetic profile of intravitreous injections of E10030 (anti-PDGF pegylated aptamer) in subjects with neovascular age-related macular degeneration, ClinicalTrials.gov identifier: NCT00569140, ClinicalTrials.gov online, http://www.clinicaltrials.gov/ct2/show/NCT00569140, accessed December 27, 2009).

A phase 2 clinical trial evaluated the efficacy and safety of E10030 administered in combination with an anti-VEGF agent for the treatment of patients newly diagnosed with wet AMD. The trial enrolled 449 patients at approximately 69 centers in North America, South America, Europe, and Israel, and E10030 (0.3 mg or 1.5 mg) administered in combination with ranibizumab injection (0.5 mg) demonstrated statistically significant improvement compared to ranibizumab monotherapy based on the primary endpoint of mean change in visual acuity from baseline at 24 weeks. The proportions of subjects gaining 15 or more ETDRS letters from baseline at the week 24 visit are 39.1% versus 34.0% in patients receiving the combination therapy (1.5 mg of E10030 and 0.5 mg ranibizumab) and ranibizumab monotherapy, respectively. E10030 exhibited a favorable safety profile and no serious adverse effects were observed for E10030 combination therapy as compared to ranibizumab monotherapy.

## 3. Anti-Immune or Anti-Inflammatory Pathways

Recently, comparative transcriptome analysis of AMD and normal human donor eyes has yielded important insights into AMD, including the molecular pathways underlying AMD's onset and progression [13]. Newman et al. [13] reported cell-mediated immune responses as the central feature of all AMD phenotypes. Therefore, addressing the role of immune response in the pathogenesis of ocular neovascularization may be a promising avenue for identifying targets for AMD treatments.

### 3.1. mTOR

Mammalian target of rapamycin (mTOR) is an evolutionarily conserved serine/threonine kinase that plays a central role in integrating environmental cues in the form of growth factors, amino acids, and energy [14]. In the study of the immune system, mTOR is emerging as a critical regulator of immune function because of its role in sensing and integrating cues from the immune microenvironment [14].

Sirolimus (previously known as rapamycin, Santen Pharmaceutical, Inc., Osaka, Kapan, and MacuSight, Inc., Union City, CA) was originally developed as a macrolide antifungal agent but was found to possess potent immunosuppressive and antiproliferative properties. Sirolimus blocks the T-lymphocyte activation and smooth muscle and endothelial cell proliferation that occurs in response to antigenic and cytokine (interleukins IL-2, IL-4, and IL-15) stimulation through either calcium-dependent or calcium-independent pathways. Sirolimus arrests cell cycle progression by direct interaction with two intracellular proteins (immunophilin FK binding protein 12 (FKBP-12) and the mammalian target of rapamycin (mTOR), a multifunctional serine-threonine kinase) [15]. In cells, sirolimus binds to FKBP-12, and the resulting sirolimus-FKBP-12 complex then binds to and inhibits mTOR [15]. Sirolimus is used to prevent rejection after organ transplantation, particularly after renal transplantation [15]. Sirolimus markedly inhibits response of vascular endothelial cells to stimulation by VEGF [16] and inhibits hypoxia-inducible factor-1*α*, a major upstream regulator of VEGF [17]. In a murine AMD model, systemic administration of sirolimus inhibited both choroidal and retinal neovascularization [18, 19].

A phase 1 study of 30 patients found that intravitreally and subconjunctivally injected sirolimus was safe and well tolerated in doses tested (phase 1/2 study of an ocular sirolimus (rapamycin) formulation in patients with age-related macular degeneration, ClinicalTrials.gov identifier: NCT00712491, ClinicalTrials.gov online, http://www.clinicaltrials.gov/ct2/show/NCT00712491). A single intravitreal administration of sirolimus (352 *μ*g) was associated with improvement in visual acuity. Additionally, patients also experienced anatomical improvements as demonstrated by a reduction in retinal thickness. Preliminary findings suggested that subconjunctival administration (sirolimus 1,320 *μ*g) was as effective as intravitreal sirolimus injection. MacuSight Inc. (Union City, CA) announces positive preliminary results from phase 1 study of sirolimus in wet age-related macular degeneration (http://www.medicalnewstoday.com/articles/98491.php, accessed December 26, 2008). Although intravitreal injection is the standard route of administration for current anti-VEGF therapies, it is uncomfortable for many patients and are accompanied by the risk of infection in a small percentage of patients. In contrast, subconjunctival injections are designed to offer physicians and patients a less invasive and more convenient procedure.

A phase 2 trial examined subconjunctival sirolimus (440 *μ*g or 1,320 *μ*g) in combination with intravitreal ranibizumab (500 *μ*g) (phase 2 study of an ocular sirolimus (rapamycin) formulation in combination with ranibizumab in patients with age-related macular degeneration (EMERALD), ClinicalTrials.gov identifier: NCT00766337).

A phase 1/2 study is underway and is examining the effect of intravitreal sirolimus in the treatment of bilateral geographic atrophy associated with AMD (ClinicalTrials.gov identifier: NCT01445548, ClinicalTrials.gov online, http://www.clinicaltrials.gov/ct2/show/NCT01445548).

Results from earlier phase 1 and phase 2 studies showed that the drug was safe and well tolerated. There was no evidence of increased intraocular pressure, inflammatory response to treatment, or indication of progression of cataracts. Patients showed improvements in visual acuity and improvements in retinal thickness.

### 3.2. TNF-*α*

Tumor necrosis factor alpha (TNF-*α*) is the prototypic ligand of the TNF superfamily. It is a key molecule that plays a central role in inflammation, apoptosis, and immune system. The anti-TNF-*α* monoclonal antibody infliximab (Remicade, Centocor, Inc., Horsham, PA, USA) is used for various inflammatory diseases, such as rheumatoid arthritis, inflammatory bowel disease, psoriasis, and noninfectious uveitis. Regression of CNV and improvement of visual acuity were reported in three wet AMD patients treated with intravenous infliximab for inflammatory arthritis [20, 21]. It has been reported that intravitreal infliximab also inhibited laser-induced CNV in rats [22]. Subsequent experiments revealed that intravitreally administered infliximab is safely increased up to 2 mg in the rabbit eye [23]. Such doses can be used in the design of future clinical trials assessing the effects of infliximab for selected patients with immune-mediated ocular conditions [23]. It has been also reported that intravitreal infliximab was administered to three patients with wet AMD, resulting in improved visual acuity and central foveal thickness on OCT [24].

A phase 1 study is now underway and is evaluating intravitreal infliximab for wet AMD, as well as for refractory diabetic macular edema (intravitreal infliximab for diabetic macular edema (DME) and choroidal neovascularization (CNV) (ITVR) (ClinicalTrials.gov identifier: NCT00695682, ClinicalTrials.gov online, http://www.clinicaltrials.gov/ct2/show/NCT00695682)).

### 3.3. Complement Component 3

Complement component 3, often simply called C3, is a protein of the immune system. It plays a central role in the complement system and contributes to innate immunity. Complement component 3 (C3) is detected in the vicinity of the drusen [25, 26]. Genetic studies revealed highly significant statistical associations between AMD and variants of C3 gene [27–30]. C3 induces VEGF expression* in vivo* and* in vitro*, and C3 gene polymorphism increases the risk of AMD [30]. Thus, inhibition of C3 suppresses complement cascade activation that could lead to local inflammation, tissue damage, and upregulation of VEGF [31].

Thus, inhibition of immune pathways may play a therapeutic role in wet AMD. POT-4 (Potentia Pharmaceuticals, Louisville, KY, USA) binds and inhibits C3 [32]. POT-4 is a synthetic peptide of 13 amino acids and “gel-like” product when injected into the vitreous. The effect is long-lasting (3 to 6 months). A phase 1 study will provide safety and tolerability information on POT-4 injected into the eyes of patients with wet AMD (Potentia pharmaceuticals' POT-4 drug candidate for age-related macular degeneration successfully completes phase 1 clinical trial. http://www.medicalnewstoday.com/articles/148725.php). POT-4 also demonstrated therapeutic efficacy in a few end-stage wet AMD patients (safety of intravitreal POT-4 therapy for patients with neovascular age-related macular degeneration (AMD) (ASaP). ClinicalTrials.gov identifier: NCT00473928. http://www.clinicaltrials.gov/ct2/show/NCT00473928). A phase 2 study to further define the safety, efficacy, and pharmacokinetic profile of POT-4 is planning.

Taken together, innovation of immunotherapy that can prevent inflammatory damage to retinal tissue may inhibit drusen formation during the early stages of AMD and afford a therapeutic to delay vision loss.

## 4. Integrin Receptor Antagonist

Integrins are transmembranous proteins that mediate the attachment between a cell and its surrounding ECM. The ligands of integrins are defined as fibronectin, vitronectin, collagen, and laminin [33]. Integrins are composed of *α* and *β* subunits that heterodimerize to produce more than 20 different receptors, capable of mediating a variety of cell responses, such as spreading and migration, control of gene expression, growth, and differentiation [34–39].

Integrin *α*v*β*5 localized at the apical surface of RPE binds to ligands in the interphotoreceptor matrix (IPM) [40] and participates in the interactions between photoreceptors and the RPE [41]. In *β*5 integrin-deficient mice, which specifically lack *α*v*β*5 integrins, retinal adhesion and phagocytosis of photoreceptor outer segments are compromised. With age, lack of *α*v*β*5 leads to declined retinal photoresponses and accumulation of autofluorescent storage bodies in the RPE [40].

In pathologic condition such as proliferative vitreoretinopathy, altered patterns of integrin expressions are associated with RPE activation, migration, and proliferation [42]. In AMD, pathological changes reduced integrin molecules and these changes may induce dissociation between the IPM and surrounding cells [43]. These changes would be predicted to decrease the supply of oxygen, nutrients, and growth factors to the cells from the choroidal or retinal vessels and inhibit the phagocytosis of photoreceptor outer segments [44].

It has been revealed that *α*v*β*1 [45], *α*v*β*3 [46], and *α*v*β*5 integrins [45, 46] were expressed in neovascular ocular tissue from patients with wet AMD. Hammes et al. [47] showed that subcutaneous injection of cyclic RGDfV peptide (antagonist of *α*v*β*3 and *α*v*β*5) remarkably prevented retinal neovascularization in a mouse model of hypoxia induced proliferative retinopathy. These results indicate the possibility that *α*v*β*3 and *α*v*β*5 integrins might be a therapeutic target for AMD [48, 49]. Additionally, the effectiveness of *α*v*β*1 and *α*v*β*5 integrin antagonists (JNJ-26076713) against ocular neovascularization has been well documented [50]. There are two therapeutic candidates to antagonize *α*v*β*1 integrin, JSM6427 and volociximab, for the treatment of AMD.

### 4.1. JSM6427

JSM6427 is a small molecule inhibitor of *α*v*β*1 integrin, which has been shown in preclinical models to inhibit retinal and choroidal neovascularization [51]. Phase I clinical trial of JSM6427 for exudative AMD has been already completed (a phase 1 safety study of single and repeated doses of JSM6427 (intravitreal injection) to treat AMD. http://clinicaltrials.gov/ct2/show/NCT00536016). Intravitreal administration of one dose of JSM6427 increased mean best corrected visual acuity in all patients.

### 4.2. Volociximab

Volociximab (Ophthotech Corporation, Princeton, NY, USA) is a chimeral monoclonal antibody specifically blocking the binding of fibronectin to the *α*v*β*1 integrin. A phase 1 study evaluated the safety of intravitreal volociximab combined with ranibizumab for neovascular AMD (a phase 1 ascending and parallel group trial to establish the safety, tolerability, and pharmacokinetics profile of volociximab (alpha 5 beta 1 integrin antagonist) in subjects with exudative age-related macular degeneration. ClinicalTrials.gov identifier: NCT00782093. ClinicalTrials.gov online. http://www.clinicaltrials.gov/ct2/show/NCT00782093). Volociximab was used in combination with ranibizumab because the volociximab was lacking an antipermeability effect. Preliminary results from 10 subjects receiving two doses of volociximab in combination with ranibizumab revealed a visual acuity improvement of 10.8 letters at nine weeks. However, these results do not separate the independent contributions from ranibizumab and volociximab [32]. Planning of a phase 2 study is underway.

### 4.3. ALG-1001

ALG-1001 (Allegro Ophthalmics, San Juan Capistrano, CA, USA) is a synthetic oligopeptide that mediates *α*5*β*1, *α*v*β*3, and *α*v*β*5 integrins. Its efficacy lasted for 90 days following one intravitreal injection in diabetic macula edema. In phase 1b clinical trial of ALG-1001 in neovascular AMD, fifteen participants who received 3 monthly injections of ALG-1001 monotherapy had an improvement of mean best corrected visual acuity more than 5 letters along with a 30% decrease in central macular thickness [52]. Since safety and well-tolerability have been observed in integrin peptide therapy, attention is now being turned to determining the best dosage and dosing regimen of ALG-1001 [53]. Phase 2a clinical studies of ALG-1001 are now going (a safety and efficacy study of ALG-1001 in human subjects with wet age-related macular degeneration, phase 2, ClinicalTrials.gov identifier: NCT00782093, ClinicalTrials.gov online, http://www.clinicaltrials.gov/ct2/show/NCT01749891).

Upregulation of integrin receptors is a downstream event to VEGF upregulation in the angiogenic cascade of AMD [54]. Therefore, it seems possible that combination of integrin peptide and anti-VEGF therapy may enhance the therapeutic effects against choroidal neovascularization. Taken together, integrin peptide therapy is an emerging new class of wet AMD treatment.

## 5. PEDF

PEDF was originally described by Tombran-Tink and Johnson [55]. It is a cell survival factor secreted by the RPE and widely expressed in central and peripheral nervous system [54–56]. PEDF is also known as an endogenous inhibitor of angiogenesis in the eye [57]. By contrast, VEGF is one of the most potent angiogenic stimulators [58]. Inappropriate expression levels of PEDF and VEGF are associated with neovascularization [59, 60]. Steinle et al. [61] characterized age-related changes in PEDF and VEGF in rat retina. Increases in VEGF and its receptor VEGFR2 and simultaneous decrease in PEDF were found in aged rats. These results suggest that normal aging retina is at increased risk for neovascular changes [61]. A critical balance appears to exist between PEDF and VEGF, with PEDF counteracting the angiogenic potential of VEGF [61]. A decrease in PEDF may disrupt this balance and create a permissive environment for the formation of CNV in AMD. Lin et al. [62] also indicate the possibilities that* PEDF* gene polymorphisms may contribute to AMD.

### 5.1. ATG003

Cigarette smoking is the strongest environmental risk factor for wet AMD [63, 64]. Recently, it has been reported that PEDF protein expression was decreased in RPE from smoker patients with AMD compared with controls [63]. The same authors also reported that nicotine, a potent angiogenic agent, increased VEGF/PEDF ratio in the RPE through nicotinic acetylcholine receptor (nAchR) [65]. These results indicate that increased VEGF/PEDF ratio might induce CNV formation in smoker patients.

In this context, a unique therapeutic eye drop for AMD treatment, ATG003, has been developed. ATG003 (CoMentis, formerly Athenagen, South San Francisco, CA, USA) is a topical mecamylamine and antagonizes nAchR pathway that mediates angiogenesis. It is the first noninvasive eye drop therapy for AMD, and phase 2 clinical trial has recently been completed (safety and efficacy of ATG003 in patients with wet age-related macular degeneration (AMD), http://www.clinicaltrials.gov/ct2/show/NCT00414206?order=1) (safety and efficacy of ATG003 in patients with AMD receiving anti-VEGF, http://www.clinicaltrials.gov/ct2/show/NCT00607750). Another phase 2 trial is evaluating the safety and efficacy of ATG003 drops in 60 patients receiving maintenance intravitreal injections of ranibizumab or bevacizumab. All patients will be treated for up to 48 weeks. This trial is currently ongoing (safety and efficacy of ATG003 in patients with AMD receiving anti-VEGF, ClinicalTrials.gov identifier: NCT00607750, ClinicalTrials.gov online, http://www.clinicaltrials.gov/ct2/show/NCT00607750).

### 5.2. AdPEDF.11

The RPE secretes both VEGF and PEDF in polarized fashion in opposing direction. Namely, secretion of PEDF is primarily apical to support the retinal neurons and photoreceptors and that for VEGF is primarily basal for maintenance of choriocallilaris and choroidal vessels [66, 67]. Sonoda et al. [66] suggested that loss of polarity of RPE in advanced AMD results in marked loss of neurotrophic and vascular support for the retina potentially leading to photoreceptor loss and blindness.

Dawson et al. [68] indicated the possibility that PEDF may be of therapeutic use, especially in retinopathies where pathological neovascularization compromises vision and leads to blindness. Recently, it has been reported that PEDF levels in aqueous humor or vitreous are decreased in patients with proliferative diabetic retinopathy (PDR), which suggests that a loss of PEDF in the human eye may contribute to the development and progression of PDR [69]. Yoshida et al. [70] previously suggested that PEDF might be used as a therapeutic target for PDR. Furthermore, animal studies also confirmed the therapeutic effect of PEDF administration for retinal neovascularization in a model of diabetic retinopathy, oxygen-induced retinopathy [71, 72], and AMD [73].

It has been shown that an adenoviral vector containing complementary DNA encoding human* PEDF*, known as AdGVPEDF.11D (GenVec, Inc., Gaithersburg, MD, USA), allows expression of large amount of PEDF in the target tissue and inhibits ocular neovascularization in murine AMD models [74]. Intravitreal injection of AdGVPEDF.11D containing the transgene for cDNA of human PEDF results in the local production of PEDF. A phase 1 single dose trial enrolled 28 patients with severe neovascular AMD [75]. The percentage of patients who had no change or improvement in lesion size at 6 months was 71% in the high-dose group versus 50% in the low-dose group. A clinical study suggested the possibility that antiangiogenic activity may last for several months (up to 6 months) after a single intravitreous injection as half of the treated lesions did not change in size from baseline [75].

Although anti-VEGF therapy (intravitreal injection of ranibizumab, pegaptanib, aflibercept, and bevacizumab) is regarded as the more effective treatment for AMD now [76], the potential therapeutics of PEDF may be positive indication for future treatment of AMD.

## 6. MMP and TIMP

MMPs are a family of zinc-containing proteolytic enzymes that are responsible for degrading ECM components and play an important role in the physiological and pathological remodeling of tissues [77]. MMPs are also thought to play a major role in cell proliferation, differentiation, angiogenesis, apoptosis, and immune defense [78]. MMPs and their inhibitors, TIMPs, colocalize in the human IPM [79] and likely play a role in physiological reconstruction and turnover of the IPM. Among the MMP isozymes, MMP-9 is essential for the development of CNV in AMD [80].

Fiotti et al. [81] found a relationship between polymorphisms in MMP-9 and neovascularization in AMD. Recently, it has been reported that* MMP-9 Rs3918242 (C→T)* single nucleotide polymorphism is found to play a significant role in the development of AMD, and the effect was more pronounced at the age of less than 65 years [82].

The circulating MMPs and TIMPs have been suggested to participate in vascular remodeling and angiogenesis [83]. However, it is still controversial whether circulating MMPs are directly linked to pathogenesis of the AMD. Chau et al. [84] firstly reported a link between raised plasma MMP-9 levels and AMD. By contrast, Zeng et al. [85] reported a link between increased levels of circulating gelatinases (MMP-2 and MMP-9) and polypoidal choroidal vasculopathy, an abnormal choroidal vasculopathy distinct from typical CNV [86] but not AMD.

In AMD lesions, it was believed that endothelial cells are activated and release MMP that destroys Bruch's membrane [87]. Conversely, it has been reported that total levels of active MMP-2 and MMP-9 were significantly reduced in Bruch's membrane-choroid preparations from AMD patients [88]. Downregulation of MMP-2 and MMP-9 may lead to the advanced pathological changes that can progress to AMD. Consistently, Ahir et al. [89] have demonstrated that administration of activated MMP-2 and MMP-9 can improve the fluid permeability of Bruch's membrane. Therefore, reactivation of the MMP pathway may be effective therapeutics in AMD patients.

The so-called “nanosecond” pulse laser uses pulse durations in the nanosecond range and restricts these heat transients to <30 *μ*m [90]. These short-pulse lasers specifically target RPE cells without damage to photoreceptors or Bruch's membrane; therefore it is suggested that they could allow therapeutic access to the entire macular region [90]. Recently, Zhang et al. [91] reported that the nanosecond laser pulse modality induces increasing of the RPE-mediated release of active MMP enzymes. Additionally, the result of one-year clinical trial using ultrashort-pulse laser application has been reported. It is interesting that a single unilateral application of the ultrashort-pulse laser to the macula of AMD patients produced bilateral improvements in macula appearance and function. This finding may be due to the result that the unilateral laser photocoagulation induces bilaterally MMP pathway activation by releasing circulating factors. Taken together, targeting the MMP activation may be useful approach to the treatment of AMD.

## 7. MTX

Recent innovations in anti-VEGF have dramatically improved the prognosis of wet AMD patients [92, 93], while some remain refractory to anti-VEGF therapy. Methotrexate (MTX)is an antifolate drug and is used in high doses to treat cancer. Primary intraocular lymphoma is treated with intravitreal MTX [94]. Low doses of MTX have also been shown to be a potent anti-inflammatory agent and are used systemically for the treatment of rheumatoid arthritis [95] or noninfectious uveitis [96]. There are several proposed mechanisms for low-dose MTX in interrupting the angiogenesis cascade at various levels [97–100].

Kurup et al. [101] reported that two wet AMD patients were refractory to anti-VEGF treatment but showed some improvement by intravitreal MTX injection (400 mg). One patient showed improved visual acuity, and both patients showed decreased serous retinal detachment and decreased perifoveal leakage 2 weeks after injection. Although these case reports indicate the potential benefits of MTX as an adjunct treatment for wet AMD patients who are resistant to the traditional anti-VEGF therapy, large-scale and long-term clinical trials are necessary to evaluate the effectiveness of intravitreal MTX.

## 8. Combination Therapy

Although the current standard of therapy is intravitreal injections of VEGF inhibitors, combination therapy with anti-VEGF therapy and various treatment modalities for exudative AMD offers another option to reduce treatment frequency. In order to further address the multifactorial pathogenesis of exudative AMD, investigators have begun to examine the combination of corticosteroids, verteporfin photodynamic therapy (VPDT), and anti-VEGF agents (Triple Therapy) [102–106]. The RADICAL (Reduced Fluence Visudyne-Anti-VEGF Dexamethasone in Combination for AMD Lesions) study is a phase 2, multicenter, randomized, single-masked study of 162 patients with CNV secondary to wet AMD (https://clinicaltrials.gov/ct2/show/results/NCT00492284). The trial is studying the following therapies.

### 8.1. 1/4 Fluence Triple Therapy

Quarter fluence verteporfin (180 mW/cm^2^ for 83 seconds to deliver 15 J/cm^2^) is followed within two hours by intravitreal ranibizumab (0.5 mg) and then intravitreal dexamethasone (0.5 mg).

### 8.2. 1/2 Fluence Triple Therapy

Half fluence verteporfin (300 mW/cm^2^ for 83 seconds to deliver 25 J/cm^2^) is followed within two hours by intravitreal ranibizumab (0.5 mg) and then intravitreal dexamethasone (0.5 mg).

### 8.3. 1/2 Fluence Double Therapy

Half fluence verteporfin (300 mW/cm^2^ for 83 seconds to deliver 25 J/cm^2^) is followed within two hours by intravitreal ranibizumab (0.5 mg).

### 8.4. Ranibizumab Monotherapy (0.5 mg)

Three verteporfin-ranibizumab combination therapies (groups 1–3) were evaluated against ranibizumab monotherapy (http://www.qltinc.com/newsCenter/2010/100622.htm). Of the three combination therapies groups with verteporfin-ranibizumab, the 1/2 fluence triple therapy group had the fewest retreatment visits compared with ranibizumab monotherapy. Through 24 months, patients in the 1/2 fluence triple therapy had a mean of 4.2 retreatment visits compared with 8.9 for patients who received ranibizumab monotherapy (*P* < 0.001). However, mean visual acuity change from baseline in the 1/2 fluence triple therapy was not statistically different from the ranibizumab monotherapy group (*P* = 0.71). The incidences of ocular adverse events were reported for 30–38% of patients in the combination therapy groups and 27% of patients in the ranibizumab monotherapy group, respectively. The adverse events in combination therapy were transient visual disturbances associated with verteporfin therapy.

Taken together, the two-year result from the RADICAL study shows not only reduction in retreatment visits, but also acceptable vision outcome and safety profile compared with ranibizumab monotherapy.

## 9. Conclusions

AMD therapy has changed dramatically over the last decade. As our understanding of the molecular mechanisms involved in AMD increases, multiple new treatments are emerging. The understanding of AMD pathogenesis could provide innovative therapeutic approaches to AMD.

## Figures and Tables

**Table 1:** Summary of neovascular AMD therapeutic agents, mechanisms, route, and phase of development.

Agent	Mechanism	Treatment	Phase
E10030	Platelet-derived growth factor (PDGF) inhibitor	iv	3

Sirolimus	Mammalian target of rapamycin (mTOR) inhibitor	iv sc	2

Infliximab	Anti-tumor necrosis factor alpha (TNF-*α*) antibody	iv	1

POT-4	Complement component 3 (C3) inhibitor	iv	2

JSM6427	Integrin receptor antagonist	iv	1

Volociximab	Anti-*α*v*β*1 integrin monocle antibody	iv	1

ALG-1001	Integrin receptor antagonist	iv	2a

ATG003	Nicotinic acetylcholine receptor (nAchR) antagonist	Eye drop	2

AdPEDF.11	Transgene for cDNA of human PEDF	iv	1

Methotrexate	Antifolate drug	iv	—

Triple combination therapy (verteporfin-ranibizumab-dexamethasone)	Targeting multiple components of choroidal neovascularization	iv (ranibizumab, dexamethasone) after VPDT	2

iv: intravitreal injection; sc: subconjunctival injection.

VPDT: verteporfin photodynamic therapy.
